# High Temperature-Induced Expression of Rice α-Amylases in Developing Endosperm Produces Chalky Grains

**DOI:** 10.3389/fpls.2017.02089

**Published:** 2017-12-06

**Authors:** Masaru Nakata, Yosuke Fukamatsu, Tomomi Miyashita, Makoto Hakata, Rieko Kimura, Yuriko Nakata, Masaharu Kuroda, Takeshi Yamaguchi, Hiromoto Yamakawa

**Affiliations:** Crop Development Division, Central Region Agricultural Research Center, National Agriculture and Food Research Organization, Joetsu, Japan

**Keywords:** α-amylase, chalky grain, developing seed, endosperm, grain quality, high temperature, rice (*Oryza sativa*), starch

## Abstract

Global warming impairs grain filling in rice and reduces starch accumulation in the endosperm, leading to chalky-appearing grains, which damages their market value. We found previously that high temperature-induced expression of starch-lytic α-amylases during ripening is crucial for grain chalkiness. Because the rice genome carries at least eight functional α-amylase genes, identification of the α-amylase(s) that contribute most strongly to the production of chalky grains could accelerate efficient breeding. To identify α-amylase genes responsible for the production of chalky grains, we characterized the histological expression pattern of eight α-amylase genes and the influences of their overexpression on grain appearance and carbohydrate components through a series of experiments with transgenic rice plants. The promoter activity of most α*-*amylase genes was elevated to various extents at high temperature. Among them, the expression of *Amy1A* and *Amy3C* was induced in the internal, especially basal to dorsal, region of developing endosperm, whereas that of *Amy3D* was confined near the ventral aleurone. These regions coincided with the site of occurrence of chalkiness, which was in clear contrast to conventionally known expression patterns of the enzyme in the scutellum and aleurone during seed germination. Furthermore, overexpression of α-amylase genes, except for *Amy3E*, in developing endosperm produced various degrees of chalky grains without heat exposure, whereas that of *Amy3E* yielded normal translucent grains, as was the case in the vector control, even though *Amy3E*-overexpressing grains contained enhanced α-amylase activities. The weight of the chalky grains was decreased due to reduced amounts of starch, and microscopic observation of the chalky part of these grains revealed that their endosperm consisted of loosely packed round starch granules that had numerous pits on their surface, confirming the hydrolysis of the starch reserve by α-amylases. Moreover, the chalky grains contained increased amounts of soluble sugars including maltooligosaccharides at the expense of starch. The integrated analyses proposed that expression of *Amy1A, Amy3C*, and *Amy3D* at the specific regions of the developing endosperm could generate the chalkiness. This finding provides the fundamental knowledge to narrow down the targets for the development of high temperature-tolerant premium rice.

## Introduction

α-Amylase (EC 3.2.1.1) is a starch-metabolizing enzyme that plays a crucial role in seed germination. In germinating seeds, α-amylase hydrolyzes endosperm starch into sugars, to nourish young seedlings (Beck and Ziegler, 1989). Cereal α-amylase genes have been classified into three subfamilies, Amy1, Amy2, and Amy3 (Huang et al., 1992). In the rice (*Oryza sativa* L.) genome, at least eight α-amylase genes, *Amy1A, Amy1C, Amy2A, Amy3A, Amy3B, Amy3C, Amy3D*, and *Amy3E*, whose nomenclatures are shown in Supplementary Table S1 and previous articles (Mitsui et al., 1996; Nanjo et al., 2004), encode peptides homologous to those of known α-amylases that contain four catalytic domains with conserved residues to form the catalytic pocket (Svensson, 1994). Because of its indispensability for seed germination, numerous studies have focused on the expression of α-amylase genes during this phase. Scutellum- and aleurone-specific induction of α-amylase expression and strict control of this by gibberellins supported the crucial role of α-amylase in triggering seed germination (Jacobsen et al., 1970; Okamoto and Akazawa, 1979; Yano et al., 2015). The gibberellin-responsive element in α-amylase gene promoters and transcription factors binding to this element have been identified (Ou-Lee et al., 1988; Kim et al., 1992; Tanida et al., 1994; Gubler et al., 1995; Lu et al., 2002). Besides germinating seeds, the expression of α-amylase genes was reported in a broad range of tissues, including leaves, roots, and calli (Huang et al., 1990).

In contrast to the pivotal role of α-amylases in seed germination, the physiological significance of these enzymes has not been well explored for seed ripening. However, it is noteworthy that the expression levels of α-amylase genes and starch-hydrolyzing activity were also elevated in immature ripening seeds, which generally accumulates and reserves starch (Baun et al., 1970; Baulcombe et al., 1987; Huang et al., 1990). According to previous investigations, wheat *α-Amy3* and rice *Amy3A* are exclusively expressed in immature grains and not in germinating seeds (Baulcombe et al., 1987; Sato et al., 2011). Rice *Amy1A, Amy3D*, and *Amy3E* were expressed in immature seeds (Huang et al., 1990). Furthermore, α-amylase protein has been detected in mature rice grains (Tsuyukubo et al., 2012). The precise expression patterns of rice α-amylases were investigated using a *GUS* reporter gene under the control of the *Amy1A* and *Amy3E* promoters in germinating seeds and mature plants (Chan et al., 1993; Itoh et al., 1995), but such knowledge is lacking in developing seeds. Therefore, a comprehensive study of spatio-temporal expression patterns of the respective α-amylase genes is needed to elucidate their physiological functions in developing seeds.

Our recent studies indicated that α-amylase induced in ripening seeds is involved in grain damage triggered by high temperature. Rice grains are efficiently filled with storage materials such as starch at temperatures of around 25°C (Yoshida and Hara, 1977), resulting in translucent grains with starch granules packed tightly in the endosperm. However, high temperature above the optimum impairs the accumulation of starch in the endosperm, resulting in immature starch granules packed loosely. Such grains contain small air spaces between the starch granules that reflect the light, thus appearing chalky (Tashiro and Wardlaw, 1991; Zakaria et al., 2002), which reduces the commercial value of the grains due to the low efficiency of milling (Fitzgerald et al., 2009). Previous transcriptomic analyses revealed that the expression levels of several α-amylase genes, *Amy1A, Amy1C, Amy3A, Amy3D*, and *Amy3E*, as well as the activity of the enzyme, were increased at elevated temperature, whilst those of starch biosynthetic genes such as granule-bound starch synthase I (*GBSSI*) and a starch branching enzyme (*BEIIb*) were decreased (Yamakawa et al., 2007; Yamakawa and Hakata, 2010; Hakata et al., 2012). These responses suggest that activation of starch degradation occurs along with the repression of starch biosynthesis in rice grains exposed to high temperature, and counteracts the deposition of starch in the maturing endosperm. Consistently, the traces of starch degradation were observed as small pits and holes on the surface of immature starch granules in the chalky part of grains ripened at high temperature (Zakaria et al., 2002; Tsutsui et al., 2013; Kaneko et al., 2016).

In order to evaluate the significance of α-amylase for grain filling, α-amylase knock-down rice plants were generated. The RNAi-mediated suppression of α-amylase genes in ripening seeds resulted in fewer chalky grains in high temperature-ripening conditions, suggesting that the activation of α-amylase by high temperature is a crucial trigger for grain chalkiness (Hakata et al., 2012). Conversely, the overexpression of α-amylase genes, *Amy1A* and *Amy3D*, in the developing endosperm produced chalky grains, even when ripened at ambient temperature (Asatsuma et al., 2006). These lines of evidence suggest that α-amylase induced in ripening grains inhibits the accumulation of starch reserves, probably through its *de novo* degradation, leading to the production of chalky grains. However, in the α-amylase-suppressed plants, sequences corresponding to highly conserved catalytic domains were used as the trigger RNA to suppress most of the α-amylase genes simultaneously (Hakata et al., 2012). Therefore, it is unclear which α-amylases function to generate chalky grains at high temperatures. Moreover, whether the site where the chalkiness occurs in high temperature conditions is consistently associated with the expression pattern of α-amylase remains to be determined, although a previous proteomic study revealed that high temperature promoted the accumulation of α-amylase proteins, namely Amy3D and Amy3E, in mature grains (Kaneko et al., 2016).

In this study, to identify α-amylase genes responsible for the production of chalky grains, we conducted the following studies using transgenic rice plants. (1) The spatial distribution of the expression of α-amylase genes at various temperatures in developing and germinating seeds were analyzed with transgenic plants harboring a *GUS* reporter gene under the control of their respective promoters. (2) The effects of respective α-amylases on grain appearance, the microstructure of endosperm starch granules, and the quantity of ingredient carbohydrates such as starch and sugars were analyzed in transgenic plants overexpressing each of α-amylase genes in the developing endosperm. The combined analyses suggested that the site-specific elevation of *Amy1A, Amy3C*, and *Amy3D* expression in the endosperm of ripening seeds is a major cause of the production of chalky grains at high temperature. Based on the distribution of expression as well as the effects of overproduction of the respective isoforms, the diverse roles of α-amylases are discussed.

## Materials and Methods

### Generation of α-Amylase Promoter-GUS and Overexpression Plants

For construction of α-amylase promoter-GUS reporter vectors, the promoters of α-amylase genes, granule-bound starch synthase I gene (*Wx*; synonymous to *Waxy* or *GBSSI*), and two seed storage protein genes, Glutelin B1 (*GluB1*) and 10-kDa prolamin (*Pro10*), were amplified from rice (*Oryza sativa* L. ssp. *japonica*, cultivar ‘Nipponbare’) genomic DNA using the primers listed in Supplementary Table S1. The PCR products for α-amylase and *GluB1* promoters, consisting of approximately 2-kb upstream fragments from the -1 of translation start sites, were digested with the restriction enzymes shown in Supplementary Table S1 (either of *Asc*I, *Bam*HI, *Bgl*II, *Hin*dIII, or *Xba*I), and were inserted into the corresponding restriction enzyme sites of a pZH2B-GUS-NOS binary vector (Kuroda et al., 2010). An approximately 0.9-kb fragment of the *Pro10* promoter was inserted into pZH2B-GUS-NOS as for the α-amylase genes, and then the NOS terminator was replaced by a 0.16-kb fragment of the *Pro10* terminator. For the *Waxy* promoter, the PCR product of the approximately 2-kb promoter region was cloned into the pGEM-T easy vector (Promega, Madison, WI, United States) to direct the 3′ end to the *Spe*I site in the vector. The plasmid was digested with *Hin*dIII and *Spe*I, and the fragment was inserted into the *Hin*dIII and *Xba*I sites of pZH2B-GUS-NOS.

For the construction of α-amylase overexpression vectors, a *Pro10* promoter and terminator were employed for developing endosperm-specific expression of α-amylases. The *Pro10* promoter was amplified from rice genomic DNA using the primer set in Supplementary Table S1. The resulting fragment has a *Hin*dIII restriction site at the 5′ end, and *Xba*I plus *Bam*HI restriction sites at the 3′ end. In addition, the *Spe*I restriction site in the flanking region of *Pro10* promoter was deleted by nucleotide substitution in the reverse primer. The pZH2B10ik binary vector (Kuroda et al., 2010) was double digested by *Hin*dIII and *Bam*HI to remove the RNAi cassette (*Pro10* promoter and aspartic protease intron), then the newly amplified *Pro10* promoter was inserted to produce the pZH2B10dT vector. Then, the coding regions of α-amylase genes, *Amy1A, Amy1C, Amy2A, Amy3A, Amy3B, Amy3C, Amy3D*, and *Amy3E*, were amplified from the corresponding full-length cDNA clones provided by the Rice Genome Resource Center at the National Institute of Agrobiological Sciences or rice genomic DNA by PCR with *Xba*I and *Kpn*I restriction sites at the respective ends using the primer sets listed in Supplementary Table S1. The fragments were cloned into the corresponding restriction sites of the pZH2B10dT binary vector. The obtained promoter-GUS vectors, overexpression vectors and pZH2B vector (Kuroda et al., 2010) (for the control lines) were introduced into *Agrobacterium tumefaciens* strain EHA101 by electroporation. Rice transformation was performed as described previously (Toki, 1997).

### Plant Materials and Growth Conditions

Rice plants were grown in plant incubators (model NC350H or LPH-410SPC; Nippon Medical & Chemical Instruments, Osaka, Japan) as described previously (Yamakawa et al., 2007). For α-amylase promoter-GUS plants and Nipponbare plants (used for soluble sugar determination), high temperature (33°C/28°C for 12-h light/12-h dark periods, respectively) and normal temperature (27°C/22°C) treatments were started 5 days after heading, as described previously (Hakata et al., 2012). In the case of α-amylase overexpression plants, the incubator was set to 27°C/22°C with the same photoperiod. α-Amylase-suppressed and control transgenic plants, which were generated in the previous study (Hakata et al., 2012), were grown and ripened at moderately high temperature (31°C/26°C). Exceptionally, non-transgenic Nipponbare plants used for the RT-PCR study were grown in an air-conditioned greenhouse set at normal temperature (27°C/25°C) until heading. Five days after heading, half of the plants were moved to another room to expose them to high temperature (33°C/29°C). At the indicated DAF, an aliquot of developing caryopses was detached from the ear, immediately frozen in liquid nitrogen, and stored at -80°C until use for the determination of GUS activity, transcript levels and enzymatic activities. Approximately 45 DAF, the rest of the caryopses were harvested, dehulled, counted, weighed and photographed.

Germinating seeds for GUS staining were collected in accordance with Itoh et al. (1995). Briefly, surface-sterilized seeds were placed in sterilized water and imbibed at high (32°C) or normal (27°C) temperature under continuous light for 0, 1, 3, or 6 days.

### GUS Assays

Histochemical analysis of GUS activity was performed in accordance with Qu and Takaiwa (2004). The caryopses were halved with a razor. Longitudinal sections of developing seeds of α-amylase promoter-*GUS* and *Waxy* promoter-*GUS* (*Wx*-*GUS*) plants were submerged in the staining solution at 37°C for 22 h, whereas seeds of seed storage protein gene (*Pro10* and *GluB1*) promoter-*GUS* plants were stained for 30 min. Longitudinal sections of germinating seeds of promoter-*GUS* plants were stained for 8 h. For 0 day seeds, 5 μg/mL of cycloheximide (Wako, Osaka, Japan) was added to the staining buffer to reduce induced expression during staining.

For fluorometric assays of GUS activity, frozen seeds (the rest of halved caryopses) were crushed using a Multi-beads Shocker (MB310; Yasui Kikai, Osaka, Japan). The resultant powder was suspended in 5 volumes (w/v) of extraction buffer (50 mM NaPO_4_, pH 7.0, 10 mM Na_2_-EDTA, 0.1% sodium *N*-lauroyl sarcosinate, 0.1% Triton X-100, 10 mM 2-mercaptoethanol). After centrifugation, 20 μL of supernatant was mixed with 180 μL of extraction buffer containing 1 mM 4-methyl umbelliferyl-β-D-glucuronide. After incubation at 37°C for 20 min, 50 μL of reaction mixture was mixed with 1.95 mL of 0.2 M Na_2_CO_3_ to terminate the reaction. Production of 4-methyl umbelliferone (4-MU) was measured using a fluorometer (FR-1500; Shimadzu, Kyoto, Japan) at an excitation wavelength of 365 nm and an emission wavelength of 455 nm. Protein concentration was determined by the dye-binding method (Bradford, 1976) using a Bio-Rad Protein Assay kit (Bio-Rad, Hercules, CA, United States) with bovine serum albumin as a standard. GUS activity was calculated as pmol 4-MU/min/μg protein.

### Quantitative RT-PCR Analysis

Total RNA was extracted from caryopses at 15 DAF for α-amylase overexpression plants, and 8, 12, 16, and 20 DAF for Nipponbare using an RNeasy plant mini kit (Qiagen, Hilden, Germany). First-strand cDNA was synthesized from each preparation of the total RNA (1 μg/reaction) using a PrimeScript RT reagent kit (Takara-Bio, Kusatsu, Japan) and an oligo-dT primer. Real-time quantitative PCR was performed using a Thermal Cycler Dice Real Time system III (Takara-Bio) with SYBR Premix Ex Taq Tli RNaseH Plus (Takara-Bio) in accordance with the manufacturer’s instructions. The PCR condition was as follows: 95°C for 10 s and 40 cycles of 95°C for 5 s and 60°C for 30 s. Gene-specific primers used for amplification are listed in Supplementary Table S1. The *UBQ5* primer pair (Jain et al., 2006) was used as an internal control. Relative gene expression was determined by the method of Pfaffl (2001).

### Determination of α-Amylase Activity

Developing caryopses harvested at 15 DAF were frozen in liquid nitrogen, and crushed using a Multi-beads Shocker. The resultant powder was suspended in a 10-fold volume of extraction buffer (10 mM HEPES-KOH, pH 7.5, 1 mM CaCl_2_) for 3 h at 4°C. The homogenate was centrifuged twice at 20,000 *g* for 5 min at 4°C. The supernatant was used to determine α-amylase activity with an α-amylase measurement kit (Kikkoman, Tokyo, Japan), which contained 2-chloro-4-nitrophenyl 6^5^-azido-6^5^-deoxy-β-maltopentaoside (N3-G5-β-CNP) as a substrate. Assay mixture containing 100 μL reaction mixture (50 μL substrate solution and 50 μL enzyme solution) and 10 μL the supernatant was incubated at 37°C for 20 min and then the reaction was terminated by the addition of 200 μL reaction stop solution. The α-amylase activity was measured by the absorbance of liberated 2-chloro-4-nitrophenol (CNP) at 400 nm. One unit of α-amylase activity was defined as the amount of enzyme needed to release 1 μmol CNP from N3-G5-β-CNP per min. The data were corrected by the protein content of the respective extracts determined by the dye-binding procedure (Bradford, 1976) with bovine serum albumin as a standard.

### Scanning Electron Microscopic Observation of Starch Granules

Rice grains were cut transversely with a razor blade, and the surface of the section was photographed. Starch granules on the fractured surface were observed using a scanning electron microscope (VE-7800; Keyence, Osaka, Japan) at an accelerating voltage of 10 kV.

### Determination of Starch Content

Brown rice grains were crushed with a hammer. Starch content of the brown rice powder was determined as described previously (Yamakawa et al., 2007). The glucose liberated by excessive digestion of starch was quantified enzymatically using an F-kit for D-glucose (Roche Diagnostics, Indianapolis, IN, United States) in accordance with the manufacturer’s instructions.

### Measurement of Soluble Sugars

Soluble sugars were extracted with 80% ethanol from ground mature grains. The extracted fraction was dried *in vacuo*, resuspended in water, and sugars were reduced by the addition of 0.25% NaBH_4_. Then, the resultant materials were dried *in vacuo*, resuspended in 0.1 N NaOH, and the soluble sugars were separated and quantified using a Dionex ICS-5000+ system (Thermo Scientific, Waltham, MA, United States) equipped with a pulsed amperometric detector and a CarboPac PA-1 column (Thermo Scientific). The separation was conducted as for determination of glucan chain length of polysaccharides in the previous report (Yamakawa et al., 2007), except that the separation was terminated at 25 min. Synthetic oligosaccharides were used as standards to confirm peak identification.

## Results

### Promoter Activities of α-Amylase Genes in Developing Seeds at High Temperature

α-Amylase genes were induced in developing seeds at high temperatures and involved in the loss of grain quality (Hakata et al., 2012). In order to investigate the spatio-temporal expression of the respective genes, transgenic plants harboring the 2-kb promoter of each α-amylase gene fused to a *GUS* reporter gene were generated and subjected to the promoter activity study. In case of *Amy1A* gene, there were two in-frame ATG codons around the deduced translational start site. The second one, reported as a preferred translation initiation point (O’Neill et al., 1990), was selected for the *Amy1A-GUS* construction. Promoters of *Pro10* and *GluB1* encoding seed storage proteins, 10-kD prolamin and glutelin B1, and *Waxy* (*Wx*) encoding granule-bound starch synthase I were selected as endosperm-specific promoters for comparison of histological localization.

**FIGURE 1:**
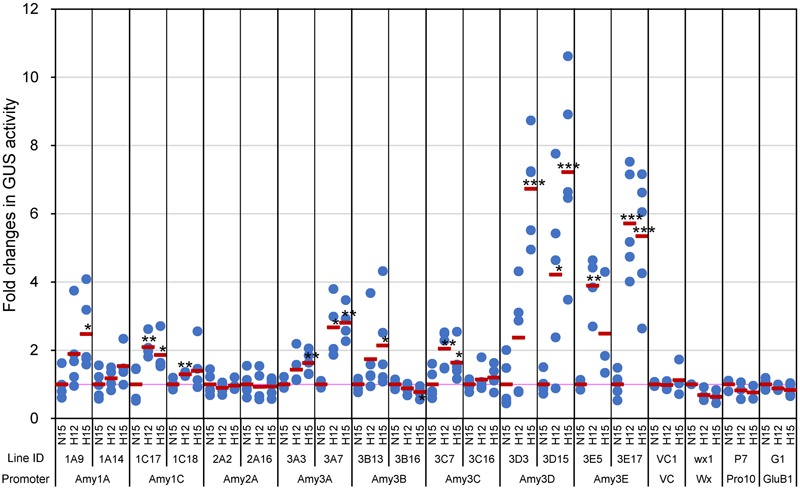
Quantitative GUS activities in developing seeds of α-amylase promoter*-*GUS plants. Developing caryopses (corresponding to T3 seeds) ripened in normal temperature condition, 27°C/22°C for day/night temperature, were sampled at 15 days after flowering (DAF) (indicated with N15), whereas those ripened in high temperature condition, 33°C/28°C, were sampled at both 12 and 15 DAF (H12 and H15, respectively). Blue dots and red bars indicate GUS activities in each individual transgenic plants, and the means of these activities in the same line, respectively. The mean value of each line at N15 was regarded as 1, and the relative fold change values are shown. Absolute values of the activity (pmol 4-MU/min/μg protein) of each line at N15 as follows, 0.052 (1A9), 0.151 (1A14), 0.036 (1C17), 0.086 (1C18), 0.042 (2A2), 0.119 (2A16), 0.020 (3A3), 0.075 (3A7), 0.030 (3B13), 0.019 (3B16), 0.023 (3C7), 0.035 (3C16), 0.353 (3D3), 0.523 (3D15), 0.085 (3E5), 0.253 (3E17), 0.014 (VC1), 0.142 (wx1), 78.283 (P7), and 71.877 (G1). Asterisks denote significant differences compared to N15 in each line, as determined by the Student’s *t*-test. ^∗^*P* < 0.05; ^∗∗^*P* < 0.01; ^∗∗∗^*P* < 0.001.

T3 transgenic plants, which were homozygous for the transgenes, were exposed to high or normal temperatures from 5 DAF. Considering that plants ripening at high temperatures matured faster than those ripening at ambient temperatures (Yamakawa et al., 2007), developing seeds were collected from the plants at 12 DAF as well as 15 DAF in the high temperature treatment, because grains at these mid-milky stages are most sensitive to high temperature in terms of grain chalkiness (Tashiro and Wardlaw, 1991). Three to five plants of respective transgenic lines were analyzed per each plot; normal temperature (27°C/22°C for day/night temperatures, respectively) at 15 DAF (N15) and high temperature (33°C/28°C) at 12 DAF and 15 DAF (H12 and H15, respectively), and at least three developing seeds were collected from each plant and cut longitudinally. Then, half of the seeds were used for measurement of GUS activity using the fluorometric assay, whereas the rest of the halved seeds were used for the histochemical analysis by GUS staining as described below. Although GUS activities of α-amylase*-GUS* plants were much lower than that of *GluB1*- and *Pro10*-*GUS* plants (see the legend of **Figure 1**), the promoter activities of *Amy1C, Amy3A, Amy3D*, and *Amy3E* increased in response to the high temperature in both of analyzed lines (**Figure 1**). Those changes agreed with the increased accumulation of Amy3D and Amy3E in the mature chalky grains after exposure to high temperature (Kaneko et al., 2016). *Amy1A, Amy3B*, and *Amy3C* also exhibited a significant increment by the elevated temperature in one of two lines examined. Considering the high temperature-induced expression of *Amy1A* in the accompanying transcriptome experiment using wild-type Nipponbare plants (Supplementary Figure S1) and our previous analysis (Hakata et al., 2012), expression of the endogenous *Amy1A* would be up-regulated by high temperature. On the other hand, no consistent induction was observed for the *Amy2A* promoter, whereas those of *Wx* and *Pro10* were slightly decreased. These profiles of α-amylase promoter activities were almost consistent with the transcript levels of the respective genes in Nipponbare plants as determined by quantitative RT-PCR (Supplementary Figure S1), confirming that the presented data reflect the expression patterns of the corresponding native promoters.

### Developing Endosperm-Specific Expression Pattern of α-Amylases

**FIGURE 2:**
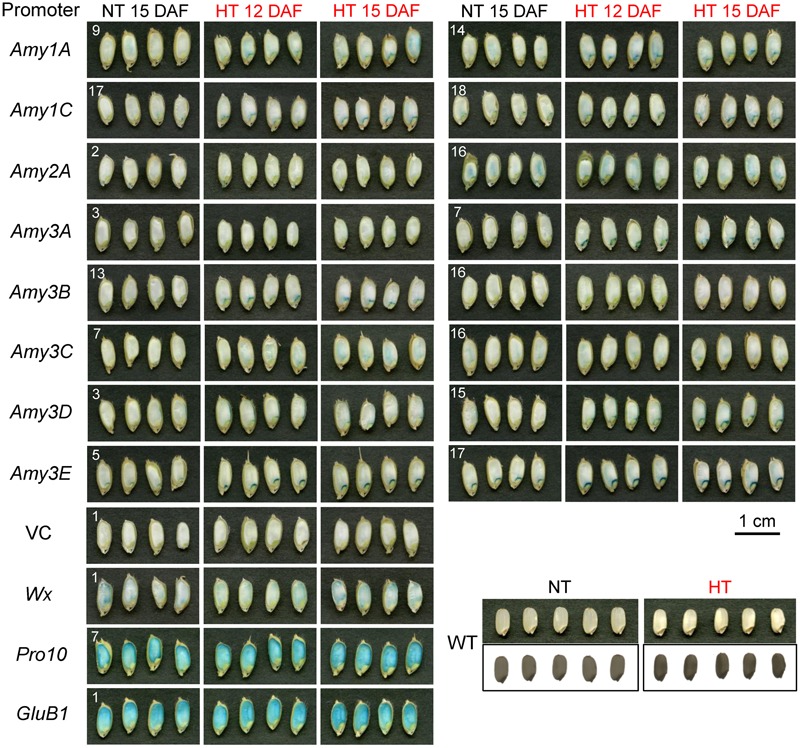
Histochemical GUS staining of developing seeds of α-amylase promoter*-*GUS plants. Two independent lines of T2 transgenic plants harboring respective α-amylase promoter-reporter chimeric genes were ripened as described in the legend of **Figure 1** (NT and HT for 27°C/22°C and 33°C/28°C plots, respectively), and the developing T3 seeds were sampled at the indicated DAF. Longitudinal sections stained for 22 h (α-amylases, *Wx* [granule-bound starch synthase I], and vector control [VC]) or 30 min (*Pro10* [10-kDa prolamin] and *GluB1*[glutelin B1]) are shown. The mature grains of Nipponbare WT, which were ripened under the same conditions, are shown in the right lower panel. The bar indicates 1 cm.

Grain chalkiness occurs in endosperm. To examine whether the expression of α-amylases coincides with the chalkiness, the location of expression of α-amylase genes was tested by histochemical staining with the same set of samples as the above quantitative experiment. The promoter activities of *Amy3E*, and weakly *Amy1C, Amy3A*, and *Amy3B*, were exclusively detected in the endosperm adjacent to the scutellum, although one line of Amy3A caryopses, line 3, presented very weak staining, and all of them were increased in response to high temperature (**Figure 2**). *Amy3D* showed a unique pattern of expression in proximity to the ventral aleurone layer, and its expression was enhanced and extended to the area surrounding the scutellum by elevation of the temperature. It was noteworthy that *Amy1A* and *Amy3C* were expressed at low levels in the internal part of endosperm at normal temperature. When the temperature rose, their expression extended from the basal to the dorsal part of the endosperm. No obvious change after the temperature shift was observed for *Amy2A* in this experiment. On the other hand, the promoter activities of *Wx, Pro10*, and *GluB1* were detected throughout in endosperm despite ripening temperatures. These expression changes in the intensity of α-amylase genes were almost consistent with the profile of the above quantitative measurement (**Figure 1**).

Because the unique endosperm-localized expression of α-amylase genes was revealed during seed ripening, we compared their histological expression profiles between developing and germinating seeds. In seeds germinating at 27°C, all the α-amylase genes, except for *Amy3A*, exhibited strong promoter activities around the scutellum or aleurone after imbibition (Supplementary Figure S2), as reported previously for *Amy1A* (Itoh et al., 1995). However, no obvious staining was detected in the inner part of the endosperm during germination. We also determined whether high temperature induced GUS activity in germinating seeds or not. *Amy1C* alone exhibited intensified expression in response to high temperature (32°C), and weak GUS activity appeared in the vicinity of the embryo of *Amy3A-GUS* seeds only at 6 days after imbibition. Compared with the influences by high temperature in developing seeds (**Figure 2**), little effect on the induction of GUS activity was observed in germinating seeds. Taken together, the induction of α-amylase expression by high temperature was a unique phenomenon observed in developing seeds, and the endosperm-localized expression of *Amy1A* and *Amy3C*, and *Amy3D* coincided with the site where the grain chalkiness occurred.

### Transgenic Rice Plants Overexpressing Each α-Amylase Gene in the Developing Seed

**FIGURE 3:**
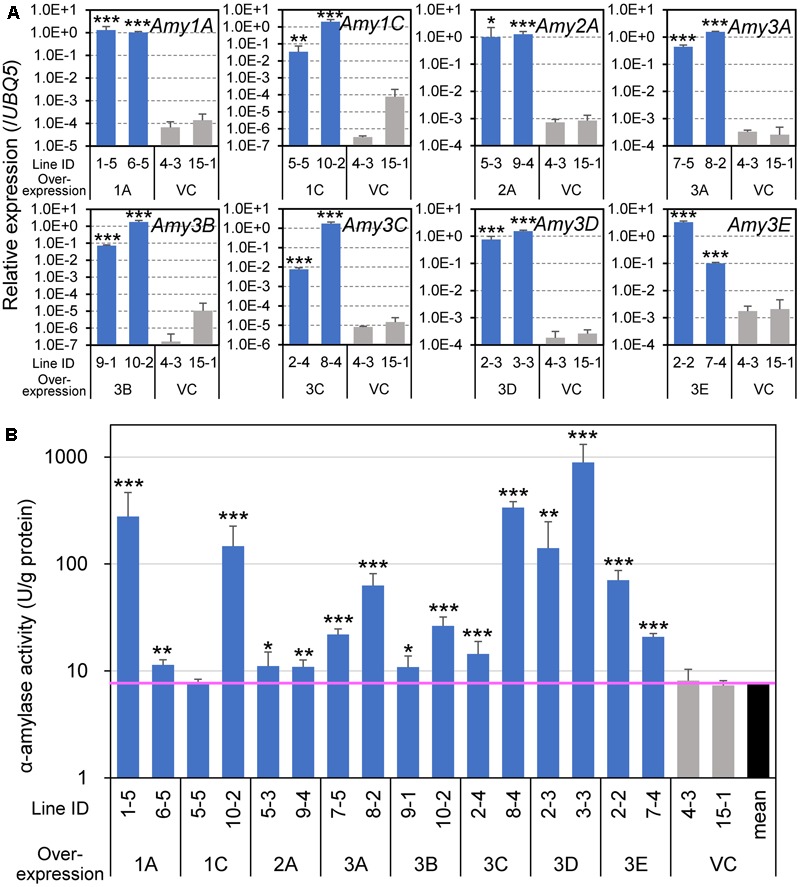
Expression of α-amylase genes and enzyme activity in developing seeds of α-amylase-overexpressing plants. **(A)** Transcript levels determined by quantitative RT-PCR analysis. Total RNA was extracted from caryopses at 15 DAF and analyzed. A constitutively expressed gene, *UBQ5*, was used as an internal control. **(B)** α-Amylase activity. Enzyme extracts from 15 DAF caryopses were used for the *in vitro* activity assay. The vertical axis uses a logarithmic scale. The pink horizontal line indicates the mean value of vector control (VC) plants. Asterisks indicate significant differences compared with VCs (VC 4-3 and 15-1), as determined by Student’s *t*-test. ^∗^*P* < 0.05; ^∗∗^*P* < 0.01; ^∗∗∗^*P* < 0.001. Bars indicate standard deviations of three independent plants.

To evaluate the influence of the expression of α-amylase in developing endosperm on grain appearance and components, we generated transgenic plants overexpressing each of the eight α-amylase genes. Because the *Pro10* gene, *Pro10*, is expressed the entire area of the developing endosperm, and especially intensively in its outer part (**Figure 2**), as reported previously (Qu and Takaiwa, 2004), we took advantage of its promoter for the overexpression of α-amylase isoforms. Two independent T3 lines, which were homozygous for the transgenes, were arbitrarily selected, and ripened at normal temperature (27°C/22°C). To confirm the enhanced expression of α-amylase genes, mid-ripening (15 DAF) seeds were examined for transgene expression levels and enzyme activity. Although the transcript levels of the introduced gene varied among lines, the expression levels of all overexpression lines were remarkably higher than that of VC lines (**Figure 3A**). An *in vitro* enzyme assay using an oligosaccharide substrate confirmed that the seeds of these transgenic plants, except for *Amy1C* expressor 5-5, contained significantly higher α-amylase activity than the VC plants (**Figure 3B**).

### Production of Chalky Grains by Overexpression of α-Amylases But Not *Amy3E* in Developing Endosperm

**FIGURE 4:**
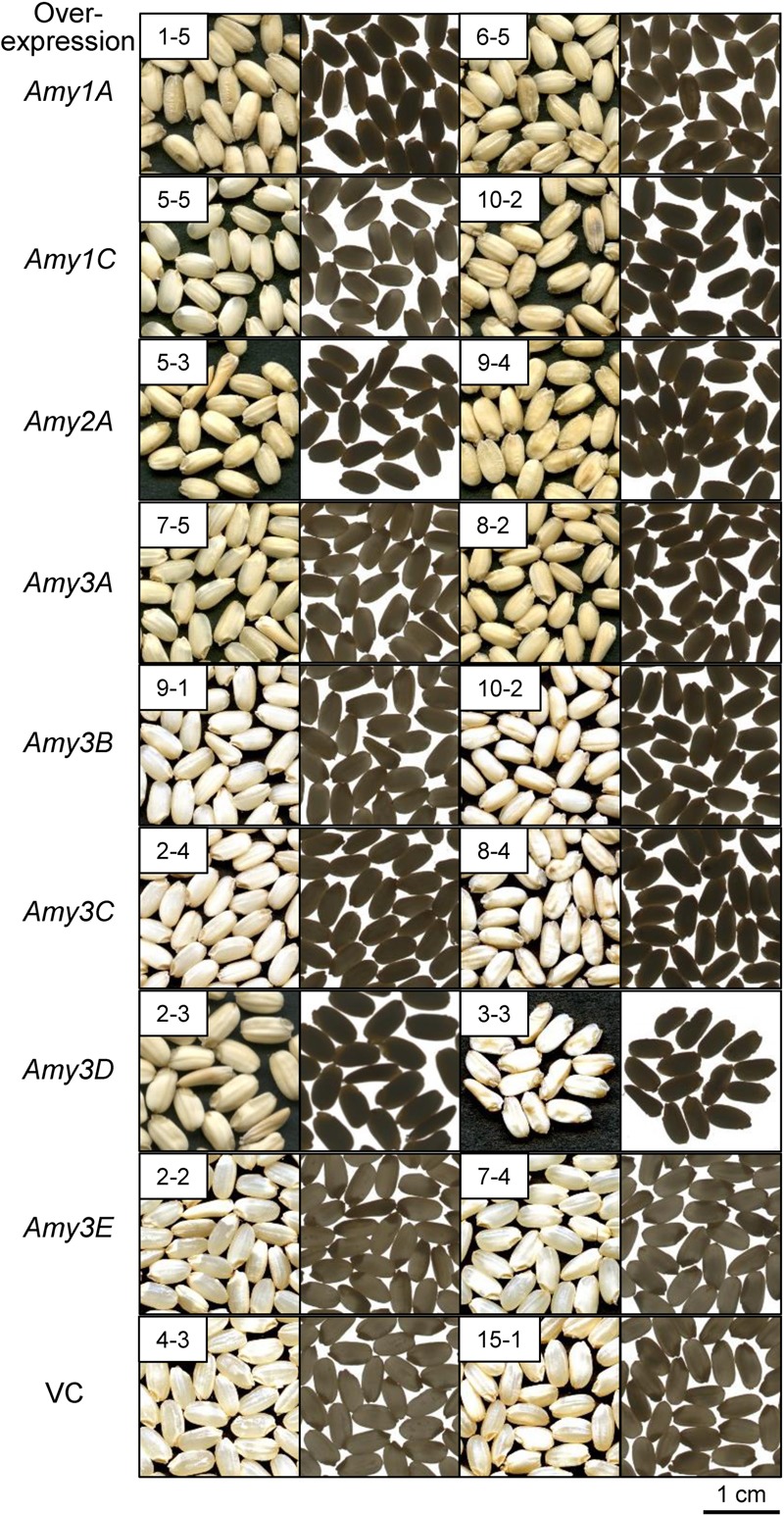
Appearance of α-amylase-overexpressing grains. Two independent lines of T3 transgenic plants were ripened at normal temperature (27°C/22°C). Mature grains obtained from the plants are shown. Images were captured using reflected light (left panel with black background) and transmitted light (right panel with white background) for each transgenic line. VC indicates vector control lines. Bar = 1 cm.

Grains overexpressing α-amylase genes other than *Amy3E* appeared chalky, even when they were ripened at normal temperature (**Figure 4**). The degrees of chalkiness were almost correlated with the intensity of enzyme activity. It was noteworthy that Amy3E-overproducing grains remained translucent, as did VC grains, even they contained high levels of α-amylase activity (**Figure 3**). One *Amy3E*-expressing line, 2-2, generated a small area of turbidity in the basal part of the endosperm near the embryo.

**FIGURE 5:**
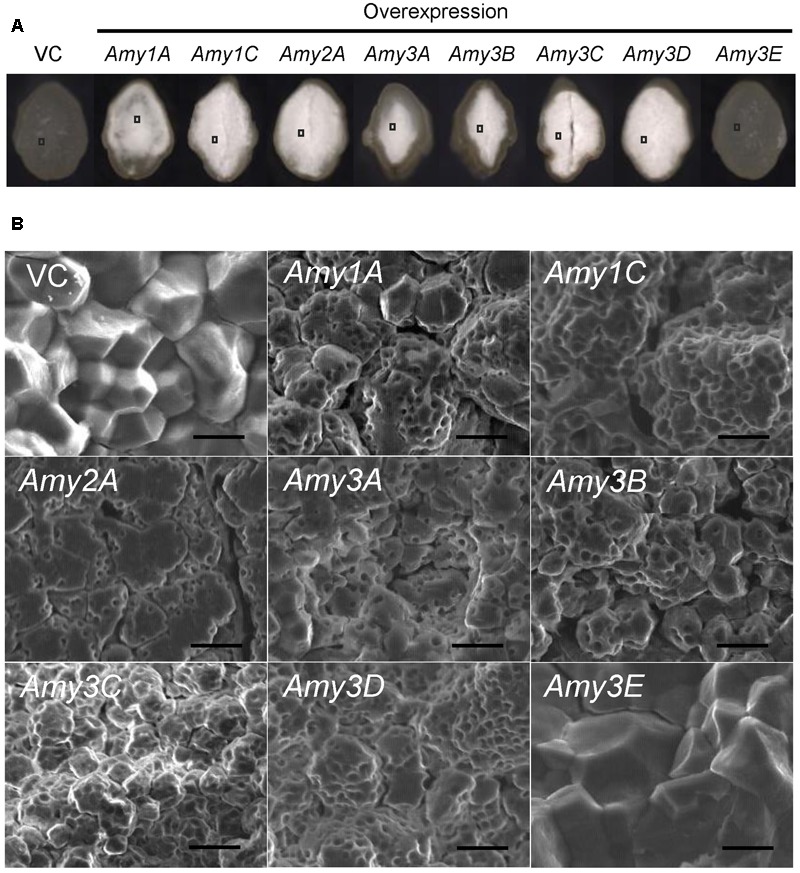
Starch granule morphology of grains of α-amylase-overexpressing plants. **(A)** Transversal cross-section of mature grains. **(B)** Scanning electron microscope (SEM) image of the area indicated by the square in **(A)**. Bar = 10 μm.

Previous reports demonstrated that the chalky appearance of grains was often attributed to the microstructure of the starchy endosperm. In the chalky area, starch granules are not completely packed, and air spaces remain among the granules and scatter light, thus leading to the chalky appearance (Tashiro and Wardlaw, 1991; Zakaria et al., 2002). To investigate the mechanism underlying the chalkiness of the α-amylase-overexpressing grains, transversal sections of mature grains were inspected using a scanning electron microscope. Despite the severity of chalkiness; chalky in the central part of the endosperm (*Amy1A, Amy3A*, and *Amy3B*), chalky in the whole endosperm (*Amy1C, Amy2A*, and *Amy3D*), or shriveled (*Amy3C*) (**Figure 5A**), all chalky parts were composed of loosely attached round granules, and numerous small pits were observed on the surface of the granules, whereas translucent endosperms of the *Amy3E* expression and VC lines were densely packed with polygonal granules (**Figure 5B**). These images implied that *de novo* degradation of starch granules occurred due to the increased α-amylase activity in developing endosperm. However, Amy3E had a peculiar feature and does not decompose starch granules in the endosperm.

### Starch Consumption in α-Amylase-Overexpressing Grains

**FIGURE 6:**
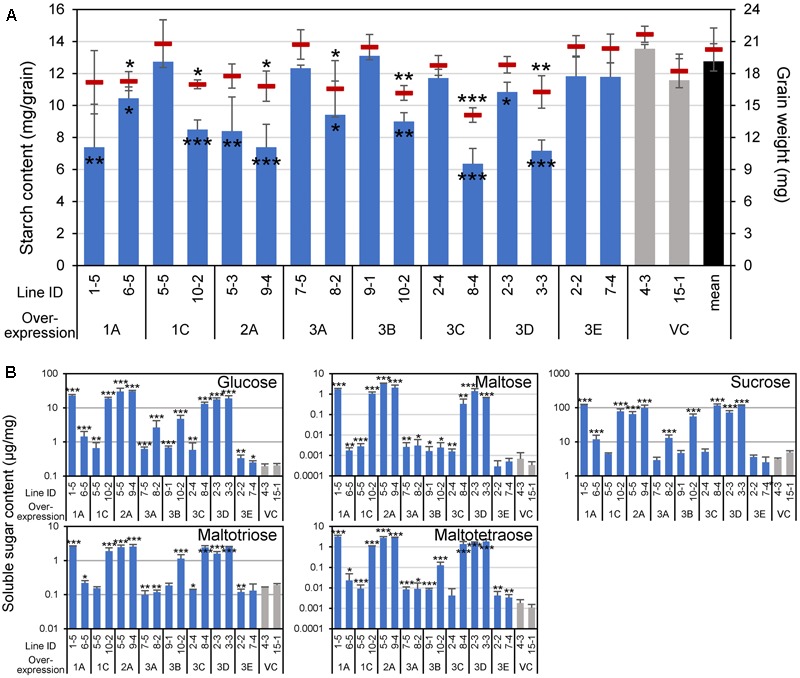
Grain weight and carbohydrate contents of α-amylase-overexpressing grains. **(A)** Starch content (mg/grain) and grain weight (mg) are indicated using vertical bars (left axis) and red short horizontal bars (right axis), respectively. Black, gray, and blue bars represent the mean starch contents of all vector control (VC) lines, each VC line, and each α-amylase-overexpressing line, respectively. **(B)** Soluble sugar contents. Contents of glucose, maltose, sucrose, maltotriose, and maltotetraose were quantified. Asterisks indicate significant differences compared with VC lines (4-3 and 15-1), as determined using Student’s *t*-test. ^∗^*P* < 0.05; ^∗∗^*P* < 0.01; ^∗∗∗^*P* < 0.001. Error bars indicate standard deviations of three independent plants.

Because the cereal endosperm is largely occupied by starch, the decomposition of starch granules by α-amylases could reduce grain weight as well as starch content, and might liberate soluble sugars. As expected, the starch content per single grain was reduced in the lines overexpressing α-amylases, other than *Amy3E*, and the grain weight was also decreased accordingly in cases where the activity was high (**Figure 6A**, compare with **Figure 3B**), probably due to the consumption of starch. Furthermore, soluble sugars, glucose, maltose, sucrose, maltotriose, and maltotetraose, the latter two of which would be exclusively produced by degradation of long glucan chains such as starch, accumulated in an enhanced activity-dependent manner (**Figure 6B**). These alterations in carbohydrate content supported the *de novo* degradation of starch by α-amylase expressed in the developing endosperm.

## Discussion

We found previously that elevated expression of α-amylases in the developing endosperm plays a crucial role in high temperature-triggered grain chalkiness. This finding shed light on potential means for the improvement of the quality of rice grains ripened under such unsuitable conditions by manipulation of the expression or activity of α-amylases (Yamakawa et al., 2007; Hakata et al., 2012). Rice genome carries at least eight functional α-amylase genes. Recently, it has been reported that an Arabidopsis α-amylase, AMY3, would be involved in the starch biosynthesis in green leaves, particularly in the removal of aberrantly branched glucan chains and the initiation of starch granules by degrading premature glucan primers (Streb et al., 2008; Seung et al., 2016). However, a conserved motif-based alignment survey using SALAD database (Mihara et al., 2010) revealed that rice genome does not carry its ortholog and all of rice α-amylases characterized in this study belong to AMY1-type (Supplementary Figure S5), suggesting that rice α-amylases might have functions which are different from those of AMY3. Identification of the α-amylase(s) that contribute most strongly to the production of chalky grains could accelerate efficient breeding to manage this unfavorable characteristic. The candidates responsible for the production of chalky grains were thought to meet the following criteria; (i) they would be expressed in the developing endosperm, especially at the same site as chalkiness occurs, and (ii) overexpression of the candidate genes in ripening grains would lead to a chalky appearance. Therefore, we investigated the spatial expression pattern in developing seeds with the promoter-reporter transgenic plants, and evaluated the consequence of enhanced production of the respective isoforms on grain appearance and carbohydrates with endosperm-specific overexpression transgenic plants. The integrated analyses proposed that expression of *Amy1A, Amy3C*, and *Amy3D* at the specific sites of endosperm could generate chalkiness, although complementary experiments using plants with suppressed expression of each of eight α-amylase genes are necessary to further strengthen this hypothesis. This finding will provide fundamental knowledge to narrow down the targets for the development of high temperature-tolerant rice plants.

Previous field experiments reported that the sites within the grain where chalkiness appear depended on the period when the developing caryopses were exposed to high temperatures. Exposure of rice plants in the early to middle ripening stages (around 4–12 DAF) induced milky-white grains, which have entirely chalky endosperm, whereas exposure in the late stage (16 DAF) produced white-back grains, which have chalkiness in the dorsal part of the endosperm (Tashiro and Wardlaw, 1991). In the transcriptome analysis in the present study (Supplementary Figure S1) and those of our previous report (Hakata et al., 2012), *Amy3C* and *Amy1A* transcripts were more abundant in the early and late stages of ripening in high temperature conditions, respectively, compared with those in normal conditions. Considering such expression durations, Amy3C and Amy1A could play major roles in the production of milky-white and white-back grains, respectively, although the expression of both at the elevated temperature covered wider areas of the internal endosperm, especially in the basal to the dorsal part (**Figure 2**). On the other hand, the distribution of *Amy3D* expression implied involvement in the production of white-belly grains, which contain chalkiness in the ventral part of the endosperm. In agreement with a previous study (Asatsuma et al., 2006), overexpression of *Amy3D* in the endosperm also induced chalky grains, supporting a relationship between *Amy3D* expression and the chalky appearance. Although the effect of high temperature on the generation of white-belly grains has remained elusive, Amy3D might be involved in the chalkiness at this area in the case of the generation of milky-white grains.

In the present study, increased expression of most α-amylases, but not *Amy3E*, produced various types of chalky grains (**Figure 4**), even when ripened at ambient temperature. Generally, chalky grains are produced when cereal endosperm is not completely filled with starch granules and remaining air spaces scatter the light. Therefore, a direct factor in triggering grain chalkiness is thought to be the imperfect deposition of starch in the endosperm. The chalky grains of α-amylase-overexpressing plants contained small decayed starch granules with numerous small pits on their surface showing traces of decomposition (**Figure 5**) and contained smaller amounts of starch than the control plants in an α-amylase activity-dependent manner (**Figure 6**), clearly supporting the idea that *de novo* degradation of starch has occurred. Similar small pits on loosely packed starch granules were observed in the chalky part of grains ripened at high temperatures (Zakaria et al., 2002; Tsutsui et al., 2013; Kaneko et al., 2016), suggesting that starch granules are degraded by α-amylases other than Amy3E in the developing endosperm in a similar manner when they are exposed to high temperature. Translocation of α-amylases into amyloplast, where starch is synthesized, is necessary to decompose starch granules. α-Amylases contain signal peptides, which lead the proteins to secrete to the outside of cells or target to specific compartments. It is noteworthy that transportation of Amy1A into plastid through Golgi apparatus secretory pathway was observed (Kitajima et al., 2009). It remains to be determined whether other α-amylases are targeted to the plastid in a similar manner.

Amy3E showed a unique characteristic with respect to the impact on grain appearance. Its overexpression neither produced chalky grains nor generated decomposed starch granules, but instead yielded normal translucent grains (**Figure 4**), even though they contained elevated levels of α-amylase activity (**Figure 3**). Similarly, wheat AMY3 overexpression failed to decompose starch during ripening due to posttranslational regulation (Whan et al., 2014). However, the failure of starch degradation by Amy3E would likely arise from its peculiar substrate preference. In our previous study, Amy3E, expressed in either rice endosperm or *Escherichia coli*, presented high activity with the oligosaccharide-based substrate, which was the same as that used in this study, but no or very low activity with long glucan chain-rich intact starch, whereas other isoforms such as Amy1A efficiently digested intact starch (Yamakawa et al., 2017). Such inefficiency in digesting long chain glucans could be the most likely reason why *Amy3E* overexpression failed to produce chalky grains. One *Amy3E*-overexpressing line, 2-2, out of two analyzed lines, occasionally produced grains with restricted chalkiness at the basal part of grains near the scutellum (**Figure 4**). In this region, other α-amylases are expressed at low levels during ripening without heat stress (**Figure 2**). Such background levels of activity might decompose starch partially and trim its glucan chains, which could permit the degradation of shortened glucans by Amy3E, thus leading to the chalky appearance in this area. Although Amy3E has some unique amino acid residues, Trp299 and Met300, at the surface binding site, which might influence the interaction with carbohydrate substrates (Cockburn et al., 2014), the molecular mechanism underlying such differences in substrate specificity remain to be determined.

Production of soluble sugars, namely glucose, maltose, sucrose, maltotriose, and maltotetraose, was apparent at the expense of starch by overexpression of α-amylase genes other than *Amy3E* (**Figure 6**). Accumulation of oligosaccharides such as maltotriose and maltotetraose would be probably due to starch degradation by the enzyme, because α-amylase is a typical endoglucanase, which produces oligosaccharides. In previous metabolomic studies, high temperature promoted the accumulation of glucose and sucrose in the mature grains (Yamakawa and Hakata, 2010; Kaneko et al., 2016). However, it was unclear whether such accumulation originated from the decomposition of starch or reduced consumption for starch biosynthesis. We also observed significant increases in maltotriose and maltotetraose as well as glucose and sucrose in non-transgenic developing seeds after exposure to elevated temperature (Supplementary Figure S3), and such an increase in glucose, sucrose, and maltotriose was attenuated in α-amylase-suppressed plants (Supplementary Figure S4). These lines of evidence further support that *de novo* degradation of starch by α-amylase occurs at high temperatures.

It is well-known that α-amylase expression levels are controlled by gibberellins during seed germination (Jacobsen et al., 1970; Okamoto and Akazawa, 1979; Yano et al., 2015). However, our previous analysis revealed that gibberellin contents in developing caryopses exposed to high temperature were similar to plants exposed to the normal temperature (Hakata et al., 2012). In addition, high temperature-induced enhancement of α-amylase gene expression was observed in developing seeds (**Figure 2**), but it was not apparent in germinating seeds (Supplementary Figure S2). Therefore, it was speculated that the expression of α-amylase genes in developing seeds and its enhancement by high temperature might be regulated, at least in part, by molecular mechanisms dissimilar to those in germinating seeds, which were independent of the effect of gibberellins. Based on our results, loss-of-function mutants of *Amy1A, Amy3C*, and *Amy3D* or their multiple mutants are expected to exhibit high temperature tolerance with less-chalky grains. However, because these α-amylases were also highly expressed in germinating seeds (Supplementary Figure S2), the mutant plants may exhibit decreased germination rates or impairment of young seedling growth. If this is the case, one possible way to avoid such untoward effects is to manipulate regulators of high temperature-specific induction of α-amylases in developing seeds. Currently, we are trying to identify such regulators by detailed analysis of the *Amy1A, Amy3C*, and *Amy3D* promoters. Furthermore, exploitation of heat-labile mutations might be an alternative strategy. Generally, seed germination is conducted at relatively low temperatures, whereas loss of grain quality occurs at higher temperatures. Given heat labile α-amylases, it is expected that starch granules in developing endosperm could be protected from high temperature-induced decay while maintaining ordinary germinability of mature seeds. Recently, we identified several temperature-sensitive mutations of Amy3D, which lost enzyme activity at lower temperature than native Amy3D (Yamakawa et al., 2017). Therefore, incorporation of such heat-labile amino acid substitutions into the innate α-amylases by genome editing could provide another tool to confer high temperature tolerance.

According to the results presented here, crucial roles of rice α-amylases in high temperature-triggered grain damage were confirmed. The expression of α-amylase genes other than *Amy2A* was induced in response to elevated temperature, and at least *Amy1A, Amy3C*, and *Amy3D* were expressed in different areas inside the developing endosperm, which corresponded to the site where chalkiness occurs. The activity of the isoforms other than Amy3E counteracted starch deposition through *de novo* decomposition of starch granules. Consistently, in previous analysis of α-amylase-suppressed grains, the extent of the decrease in chalky grains was correlated with their suppression level except for *Amy3E* (Hakata et al., 2012). To alleviate the high temperature-triggered loss of grain quality, isoforms other than Amy3E, especially Amy1A, Amy3C, and Amy3D, should be modified. Therefore, the development of high temperature-tolerant rice using mutants of these α-amylase isoforms and the search for regulators of their expression in developing endosperm are in progress to achieve the sustainable production of premium rice even with the effects of recent global warming.

## Author Contributions

HY conceived the research, and MN, TM, and HY designed experiments. MN, TM, YF, MH, RK, YN, MK, TY, and HY performed the experiments and analyzed the data. YF, MN, TM, and HY wrote the manuscript. All authors read and approved the final manuscript.

## Conflict of Interest Statement

The authors declare that the research was conducted in the absence of any commercial or financial relationships that could be construed as a potential conflict of interest. The reviewer NF and handling Editor declared their shared affiliation.

## Abbreviations

DAF: days after flowering
GUS: β-glucuronidase
Pro10: 10-kDa prolamin
UBQ5: Ubiquitin 5
VC: vector control
Wx: Waxy
