# Supplementary material for: High Temperature-Induced Expression of Rice α-Amylases in Developing Endosperm Produces Chalky Grains
Source: Front Plant Sci. 2017 Dec 6;8:2089. doi: 10.3389/fpls.2017.02089 (PMC5723670; doi:10.3389/fpls.2017.02089)
Supplement: Supplementary file 2 [file Image_1.PDF]

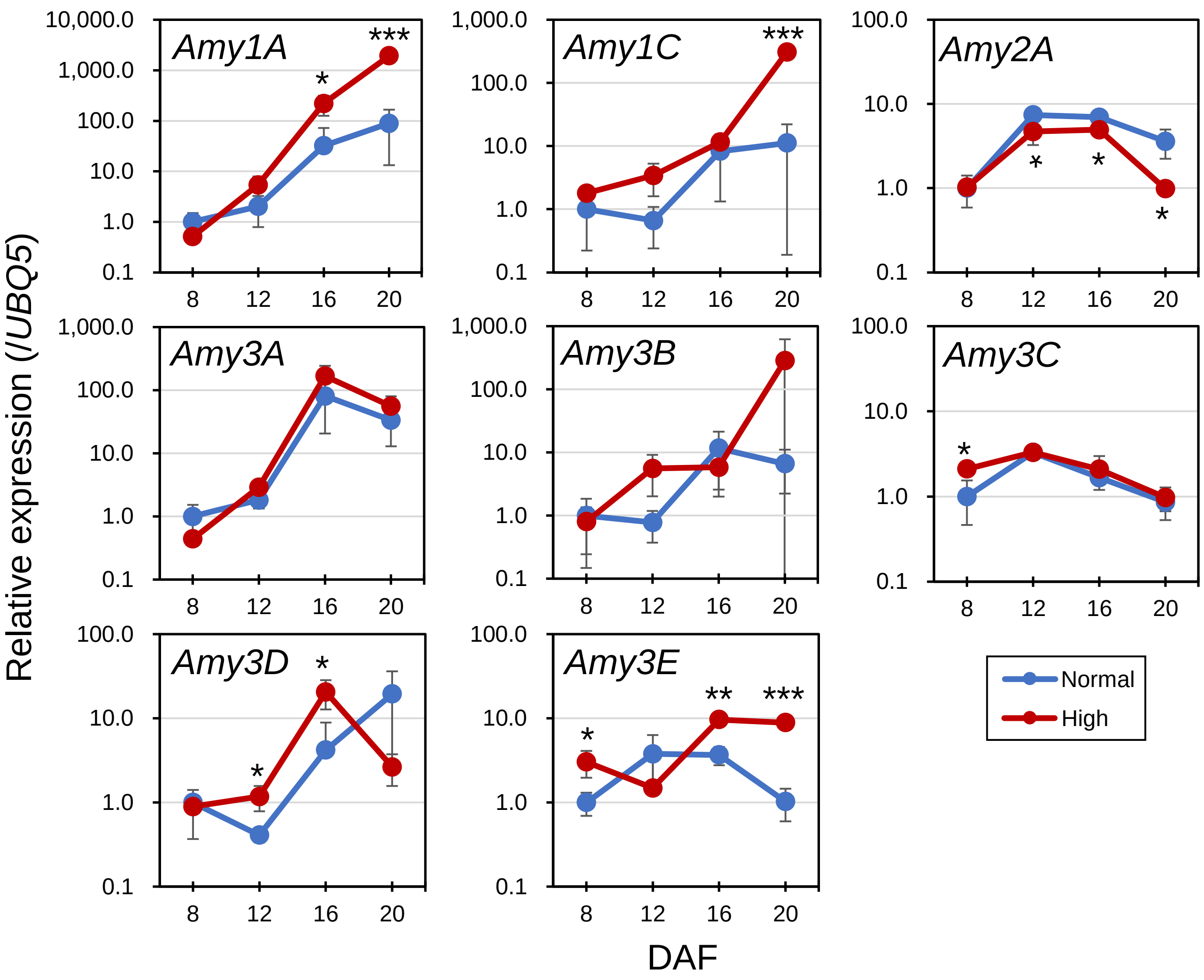

Supplementary Figure S1. Expression of α-amylase genes at normal or high temperatures in Nipponbare. Wild-type, Nipponbare caryopses were ripened at normal and high temperatures (27° C/25° C and 33° C/29° C, respectively), and total RNA was extracted at 8, 12, 16, and 20 DAF and subjected to quantitative RT-PCR. Expression levels of the normal temperature plot at 8 DAF was defined as 1, and relative transcript levels are shown. Asterisks indicate significant differences compared with the value at normal temperature at the same DAF, as determined using Student's t-test. *, $P<0.05$; **, $P<0.01$; ***, $P<0.001$. Bars indicate standard deviations of three independent samples.
