# Supplementary material for: High Temperature-Induced Expression of Rice α-Amylases in Developing Endosperm Produces Chalky Grains
Source: Front Plant Sci. 2017 Dec 6;8:2089. doi: 10.3389/fpls.2017.02089 (PMC5723670; doi:10.3389/fpls.2017.02089)
Supplement: Supplementary file 3 [file Image_2.PDF]

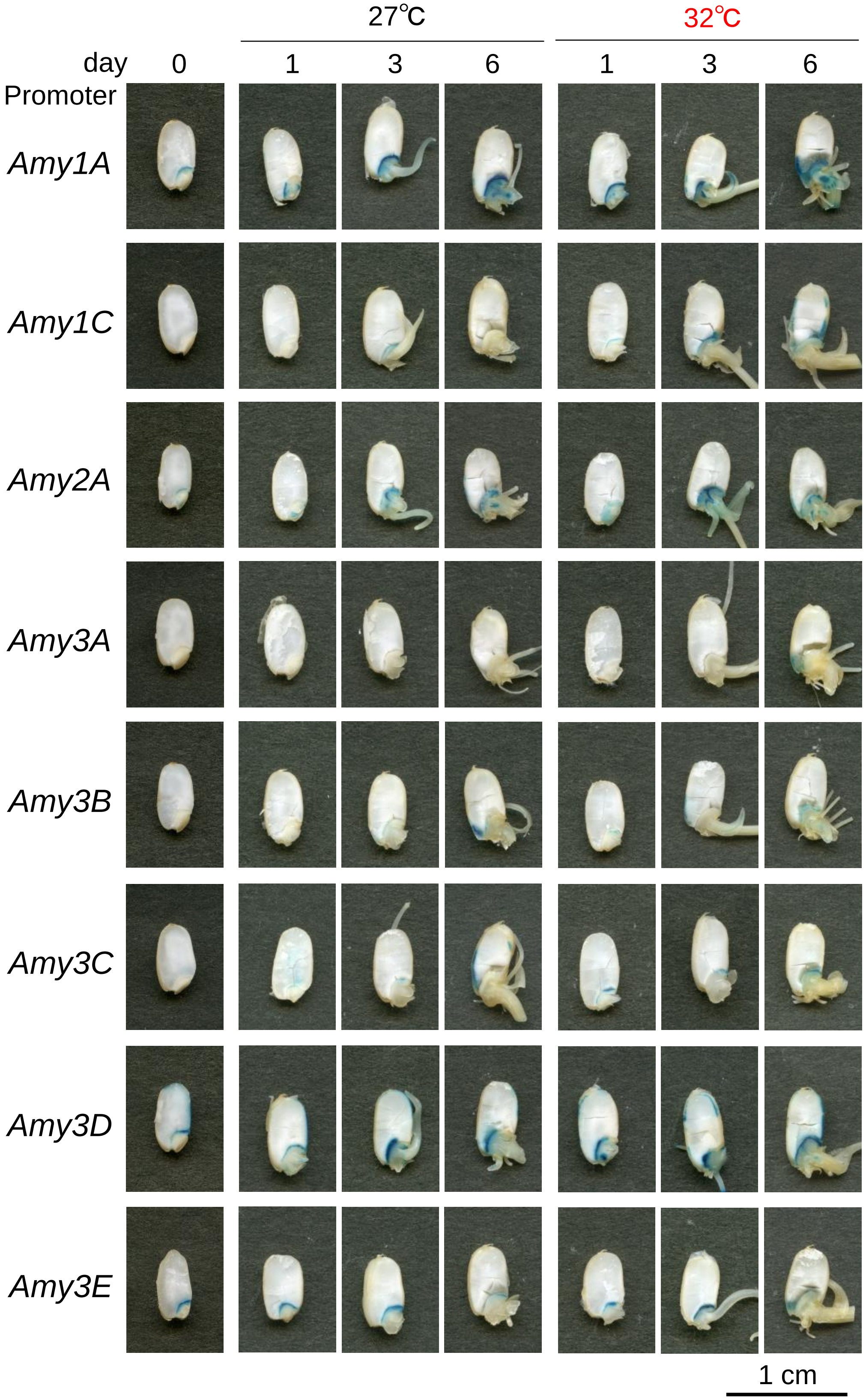

Supplementary Figure S2. Histochemical GUS staining of germinating seeds of α-amylase promoter-GUS plants. T3 seeds harboring the respective α-amylase promoter-reporter genes were imbibed for 0, 1, 3, and 6 days at normal (27° C) or high temperatures (32° C). Longitudinal sections of germinating seeds in α-amylase promoter-GUS transgenic plants are shown. In order to minimize induced gene expression during staining, 0-day seeds were stained in the presence of 5 μg/mL cycloheximide.
