# Supplementary material for: High Temperature-Induced Expression of Rice α-Amylases in Developing Endosperm Produces Chalky Grains
Source: Front Plant Sci. 2017 Dec 6;8:2089. doi: 10.3389/fpls.2017.02089 (PMC5723670; doi:10.3389/fpls.2017.02089)
Supplement: Supplementary file 4 [file Image_3.PDF]

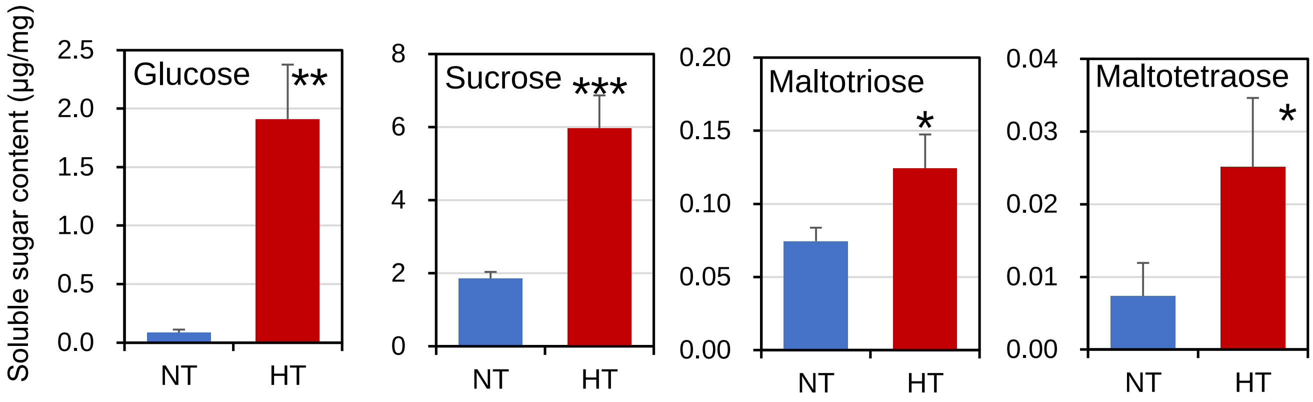

Supplementary Figure S3. Effect of ripening temperature on soluble sugar contents in the grains of wild-type plants. Glucose, sucrose, maltotriose, and maltotetraose were quantified in mature grains ripened at normal temperature (NT, blue; 27° C/22° C) and high temperature (HT, red; 33° C/28° C). Asterisks indicate significant differences compared with NT grains, as determined using Student's t-test. *, P<0.05; **, P<0.01; ***, P<0.001. Bars indicate standard deviations of three independent plants.
