# Supplementary material for: High Temperature-Induced Expression of Rice α-Amylases in Developing Endosperm Produces Chalky Grains
Source: Front Plant Sci. 2017 Dec 6;8:2089. doi: 10.3389/fpls.2017.02089 (PMC5723670; doi:10.3389/fpls.2017.02089)
Supplement: Supplementary file 5 [file Image_4.PDF]

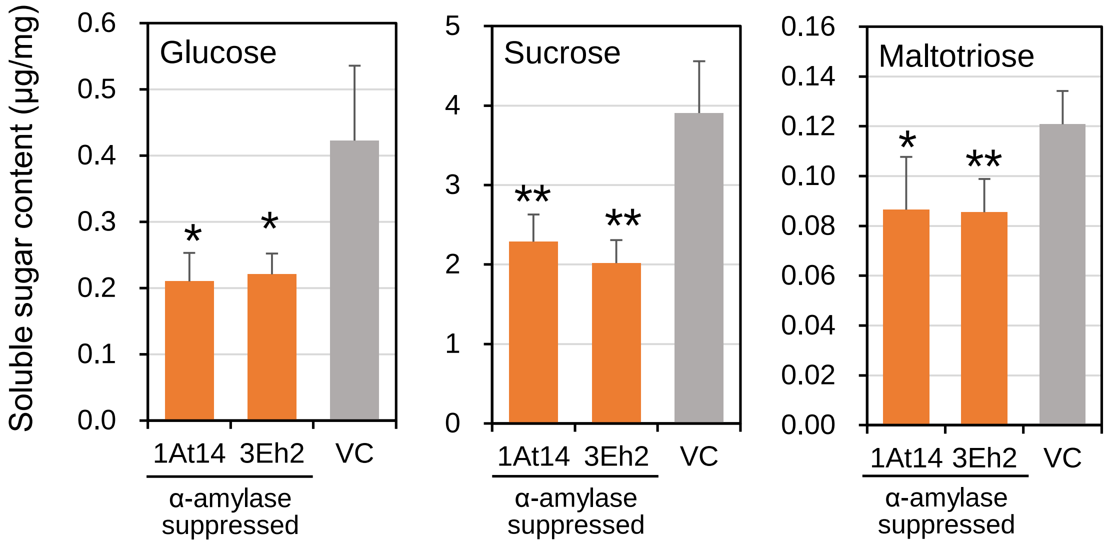

Supplementary Figure S4. Soluble sugar contents in α-amylase-suppressed grains. Grains harvested from two α-amylase-suppressed lines, which were ripened at moderately high temperature (31° C/26° C) (Hakata et al., 2012; lines 1At14 and 3Eh2), were analyzed in comparison with vector control (VC) grains. Asterisks indicate significant differences compared with VC grains, as determined using Student's t-test. *, P<0.05; **, P<0.01; ***, P<0.001. Bars indicate standard deviations of four independent plants.
