# Supplementary material for: High Temperature-Induced Expression of Rice α-Amylases in Developing Endosperm Produces Chalky Grains
Source: Front Plant Sci. 2017 Dec 6;8:2089. doi: 10.3389/fpls.2017.02089 (PMC5723670; doi:10.3389/fpls.2017.02089)
Supplement: Supplementary file 6 [file Image_5.PDF]

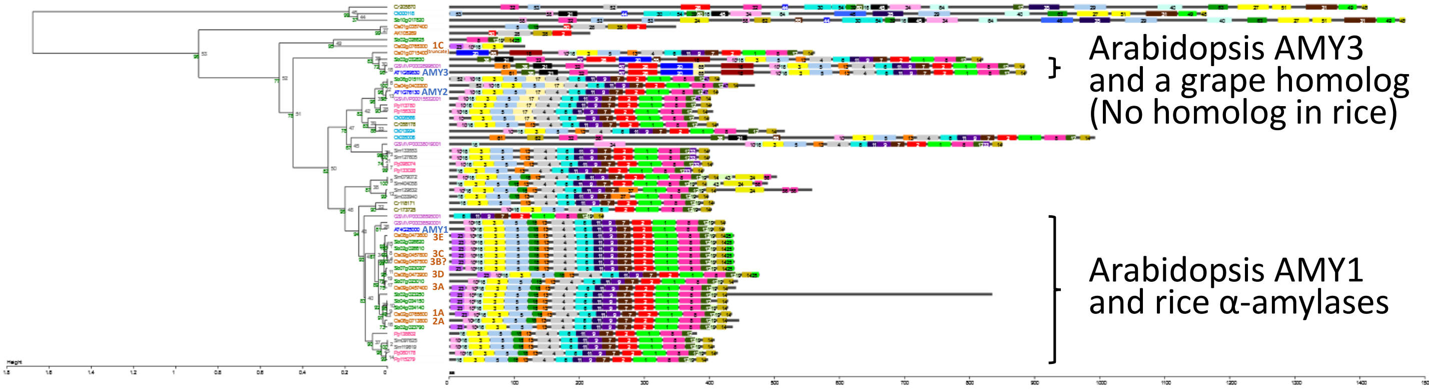

Supplementary Figure S5. A conserved motif-based alignment of plant α-amylases. Alignment of related peptide sequences was depicted by using SALAD database (http://salad.dna.affrc.go.jp/salad/) with Arabidopsis AMY3 (At1g69830) as a query. Arabidopsis and rice genes are indicated with blue and brown characters.
